# Teacher-student interaction and self-regulated learning in online higher education: a grounded theory model of interaction convertibility

**DOI:** 10.3389/fpsyg.2026.1989345

**Published:** 2026-10-13

**Authors:** Zhiwei Chen, Zhixin Liao

**Affiliations:** 1 School of Education, Minzu University of China, Beijing, China; 2 Institute of Education, Xiamen University, Xiamen, China

**Keywords:** grounded theory, interaction convertibility, online higher education, self-regulated learning, teacher-student interaction

## Abstract

**Introduction:**

Online higher education places greater demands on students’ self-regulation while making instructional support increasingly dependent on digitally mediated interaction. Although teacher–student interaction has been associated with self-regulated learning (SRL), less is known about how interaction becomes incorporated into students’ regulatory processes.

**Methods:**

Using a Straussian grounded theory approach, this study examined the online learning experiences of 30 Chinese university students through semi-structured interviews conducted between April and May 2025. A relational model was developed through the analysis of 24 interviews, followed by six additional interviews used to assess theoretical saturation.

**Results:**

Online learning conditions shaped opportunities for regulatory coupling. Interaction supported SRL when interactional input was recognized, taken up, and translated into changes in attention, understanding, strategy, or subsequent action. Resource mobilization, monitoring and reflection, and environment, time, and task management further helped stabilize this coupling across learning phases.

**Discussion:**

The study develops the concept of interaction convertibility to explain how teacher–student interaction becomes, or fails to become, a usable regulatory resource in online higher education.

## 1 Introduction

Online learning has become an established modality in higher education, extending well beyond its emergency use during the COVID-19 pandemic. This shift has altered how learning is organized: students are afforded greater flexibility in when and how they learn, but they also face stronger demands for managing attention, pacing, and learning progress with reduced face-to-face supervision (Imran et al., 2023; Xu et al., 2022). At the same time, digitally mediated environments reshape how teachers and students interact. Recent qualitative evidence suggests that productive technology-enhanced learning depends not simply on the availability of digital tools, but on relational and pedagogical practices that foster dialogue, responsiveness, learner participation, and meaningful feedback (Rashidi et al., 2025). Teacher–student interaction is also shaped by how instructional talk is organized. For example, Farangi et al. (2024) identified systematic differences in the thematic organization of classroom discourse between more and less experienced EFL teachers, highlighting teacher talk as an important component of classroom interaction. These conditions make both self-regulated learning (SRL) and teacher-student interaction central to understanding how students sustain learning online (Miao and Ma, 2022; van Dorresteijn et al., 2025).

SRL refers broadly to learners’ active regulation of their goals, strategies, attention, and learning progress (Zimmerman and Schunk, 2011). In online environments, its importance is heightened because students must initiate and sustain learning with fewer external prompts while managing distraction and fragmented learning conditions (Molenaar et al., 2023; Xu et al., 2022). Yet such regulation does not occur in isolation. Teacher–student interaction provides an important channel through which instructional and relational support becomes available to learners. Instructor feedback, clarification, reminders, and socio-emotional support can provide external resources that help students monitor understanding and adjust learning strategies (Li et al., 2023; Lim et al., 2024; Miao and Ma, 2022). The relationship may also operate in the opposite direction: students who regulate their learning more effectively may be more likely to seek help, articulate difficulties, and make productive use of feedback and interactional support (Khuder, 2025). Teacher-student interaction and SRL may therefore develop through mutually reinforcing or mutually weakening processes in online learning.

Existing research has established associations among online interaction, SRL, engagement, satisfaction, and learning outcomes, but provides less explanation of the processes through which interaction becomes part of learners’ self-regulation (Miao and Ma, 2022; Wu et al., 2023). In particular, recent studies show that feedback uptake varies across learners, feedback characteristics, and instructional contexts, and that the availability of feedback does not necessarily lead to its use in subsequent learning (Alharthi, 2025; Chen et al., 2026; Lipnevich et al., 2025). Less is known, however, about why some interactional experiences lead students to refocus attention, revise understanding, or adjust strategies, whereas others remain poorly matched to learner needs or disconnected from subsequent regulation.

Using a Straussian grounded theory approach, this study draws on in-depth interviews with 30 Chinese university students to develop a relational model of teacher-student interaction and SRL. Rather than testing a predetermined causal structure, the study examines how interaction and self-regulation become coupled or decoupled across students’ online learning experiences. Two research questions guide the analysis:

*RQ1:* How do online learning conditions shape the relationship between teacher-student interaction and self-regulated learning?

*RQ2:* Through what processes does teacher-student interaction become integrated into or remain disconnected from students’ self-regulated learning?

By addressing these questions, the study seeks to clarify the mechanism through which interactional support becomes a usable regulatory resource and to provide a process-oriented basis for the design of interaction and learning support in online higher education.

## 2 Literature review

### 2.1 Self-regulated learning in online higher education

The normalization of online and blended learning in higher education has increased the importance of learners’ capacity to regulate their own learning. Online environments provide greater flexibility in pacing, access to resources, and choice over when and where learning takes place, but they also shift a larger share of responsibility for initiating tasks, maintaining attention, monitoring progress, and managing competing demands to students themselves (Imran et al., 2023; van Dorresteijn et al., 2025). These characteristics make self-regulated learning (SRL) particularly consequential in online higher education (Prasse et al., 2024).

SRL is commonly conceptualized as a cyclical process through which learners plan learning activities, monitor their cognition and behavior during task performance, and evaluate and adjust subsequent strategies (Boekaerts et al., 2000). Recent research has increasingly emphasized that these regulatory processes are dynamic and context-dependent rather than stable learner attributes (Cristea et al., 2025; Molenaar et al., 2023). Advances in process-oriented and multimodal measurement, for example, have highlighted substantial variation in how SRL unfolds over time and across learning situations (Molenaar et al., 2023). Research on technology-based scaffolding similarly shows that external support can alter learners’ regulatory processes, although its effects depend on when and how support is provided and used (Li et al., 2023; Lim et al., 2024). In particular, Lim et al. (2024) found that the provision of scaffolds alone did not fully explain learning outcomes; learners’ actual interaction with and use of those scaffolds was also consequential.

This contextual perspective is especially relevant to online learning, where students’ regulation develops alongside social and instructional resources (Martínez-López et al., 2023; Prasse et al., 2024). Empirical evidence has linked online interaction and self-regulation with students’ engagement, while other studies have shown that SRL can mediate relationships between online interaction and learning-related outcomes such as satisfaction (Dong et al., 2024; Miao and Ma, 2022; Wu et al., 2023). These findings indicate that SRL cannot be understood solely as an internally generated capacity. Its enactment is embedded in a learning environment in which instructional guidance, feedback, and interaction can either support or constrain learners’ efforts to regulate their learning (Martínez-López et al., 2023; Prasse et al., 2024). Recent meta-analyses further confirm positive associations between SRL strategies and academic performance in online and blended higher education (Cheng et al., 2025; Zhao et al., 2025).

### 2.2 Teacher-student interaction as a regulatory resource for SRL

Online learning also changes the conditions under which teacher-student interaction occurs. Interaction that may emerge relatively spontaneously through co-presence in face-to-face settings must often be deliberately organized through synchronous sessions, discussion spaces, feedback channels, and other digitally mediated practices (Krebs-Lazendic et al., 2026). Recent qualitative evidence suggests that effective technology-enhanced learning depends substantially on pedagogical practices that create meaningful dialogue, responsiveness, student participation, and feedback, rather than on the presence of technology alone (Rashidi et al., 2025). Likewise, a systematic review of instructor participation in asynchronous online discussions found that the effects of instructor involvement varied according to the form of participation, course conditions, and learner characteristics, indicating that interaction cannot be evaluated adequately by its presence or frequency alone (Xie and Correia, 2024).

Research on teaching presence and online interaction nevertheless provides strong evidence that instructor involvement matters for students’ learning experiences (Hu and Xiao, 2025). A meta-analysis found positive relationships between teaching presence and both perceived learning and satisfaction (Caskurlu et al., 2020). At the process level, teacherstudent interaction may contribute to regulation by clarifying expectations, identifying misunderstandings, redirecting attention, providing corrective feedback, and supporting subsequent strategy adjustment (Nicol and Macfarlane-Dick, 2006; Van de Pol et al., 2010). Recent work on SRL scaffolding demonstrates that external prompts and supports can modify the sequence and organization of regulatory activities (Öztürk et al., 2025; Zheng et al., 2023). Feedback-seeking research further shows that students themselves can actively incorporate interaction into regulation by identifying gaps, initiating feedback inquiry, and strategically using feedback to determine subsequent action (Khuder, 2025).

These findings point to an important qualification: interaction may provide regulatory support, but its presence alone does not ensure that learners will interpret, take up, and use it in subsequent learning (Carless and Boud, 2018; Eggers et al., 2024; Lim et al., 2024). Research on feedback literacy similarly emphasizes that the educational value of feedback depends on learners’ capacity to interpret and use it for subsequent action (Carless and Boud, 2018; Carless and Young, 2025). Instructor participation can produce heterogeneous outcomes (Xie and Correia, 2024), and personalized scaffolding studies suggest that the effects of support depend partly on whether learners actually engage with and use the support provided (Lim et al., 2024). Quantitative studies have established associations among interaction, SRL, engagement, and satisfaction (Miao and Ma, 2022; Wu et al., 2023), but such associations provide limited insight into what happens between an interactional event and a subsequent change in learners’ regulation. This distinction is particularly important in online settings, where the effects of instructor involvement vary according to the form of interaction, course conditions, and learner characteristics, and where the presence of interaction alone does not guarantee meaningful learner engagement or regulatory uptake (Eggers et al., 2024; Xie and Correia, 2024).

### 2.3 Integrating teacher-student interaction and SRL

Taken together, the literature suggests that self-regulated learning and teacher-student interaction should not be treated as separate components of online learning (Eggers et al., 2024; Martínez-López et al., 2023). Classic accounts of SRL have long emphasized feedback as integral to monitoring and subsequent regulatory adjustment (Butler and Winne, 1995). SRL involves learners’ ongoing planning, monitoring, and adjustment of learning, while teacher-student interaction can provide external input through clarification, feedback, prompting, and academic help (Eggers et al., 2024; Öztürk et al., 2025). From this perspective, interaction may become relevant to SRL when it helps learners identify difficulties, reconsider their understanding, redirect attention, or determine subsequent action (Khuder, 2025; Li et al., 2023; Lim et al., 2024; Zimmerman, 2002). This view positions regulation as a process that develops through the interplay between learners’ own regulatory activity and the instructional and social resources available in their learning environment (Martínez-López et al., 2023).

Existing research has documented associations between interactional support and students’ self-regulation, as well as links between SRL and feedback uptake in blended and online learning environments (Chen et al., 2026; Eggers et al., 2024). However, these studies provide less insight into the process through which particular interactional inputs become incorporated into learners’ ongoing regulation. Studies have also linked online interaction and self-regulation to engagement, satisfaction, and other learning outcomes (Miao and Ma, 2022; Wu et al., 2023; Xie and Correia, 2024). Recent evidence further shows that learners do not consistently use instructional feedback once it is provided: feedback may be rejected because of ambiguity, lack of trust, or learner-related factors, and the extent of uptake can vary across interactional settings (Alharthi, 2025; Lipnevich et al., 2025). What remains less clear is how these differences in uptake unfold within students’ broader self-regulatory processes, why some interactional inputs lead to changes in attention, understanding, or learning strategy whereas others do not, and how these processes operate under the particular affordances and constraints of online learning. Addressing this gap requires a process-oriented account of the relationship between teacher-student interaction and SRL rather than another test of their statistical association.

## 3 Method

### 3.1 Research design and methodological orientation

This study employed a qualitative research design informed by Straussian grounded theory to examine how teacher-student interaction becomes linked to students’ self-regulatory processes in online higher education. A grounded theory approach was appropriate because the study aimed to develop a process-oriented explanation of an underexplored relationship rather than test predetermined associations among variables. The Straussian tradition was particularly suited to this purpose because it provides an analytical framework for examining conditions, actions or interactions, and consequences and for specifying relationships among emerging categories (Corbin and Strauss, 2015; Strauss and Corbin, 1998).

The analysis was primarily inductive and data-driven. Existing scholarship on self-regulated learning and teacher-student interaction informed the study as sensitizing concepts, directing attention to potentially relevant learning and interactional processes without determining the categories in advance (Bowen, 2006). No predefined codebook or theoretical categories were imposed on the interview data. Instead, concepts, categories, and their relationships were progressively developed through open, axial, and selective coding, accompanied by constant comparison throughout the analysis.

### 3.2 Sampling strategy, participants, and data collection

A criterion-based purposive sampling strategy was used to recruit participants with direct experience of online university learning and teacher-student interaction in digitally mediated courses (Palinkas et al., 2015). Participants were recruited through a combination of online and offline channels. Online recruitment was conducted through multiple internet-based channels, while offline recruitment involved in-person promotion and recruitment at university campuses. Eligibility required participants to have prior experience with online university learning and teacher-student interaction in digitally mediated courses. The final sample consisted of 30 university students in China, aged 18–25 years, with variation in gender, year of study, disciplinary background, and geographical region. Because age was not used as an analytical grouping variable, it is reported as a range rather than divided into arbitrary categories.

Semi-structured, one-to-one interviews were conducted between April and May 2025. Each interview lasted approximately 30–45 min and was conducted in Chinese. Both authors participated in conducting the interviews. Participants were invited to describe concrete experiences of online learning, including how they organized and adjusted their learning, when and why they interacted with instructors, how they responded to feedback or instructional support, and situations in which interaction did or did not influence their subsequent learning.

All interviews were audio-recorded with participants’ consent and transcribed verbatim. All participants provided written informed consent prior to participation. Identifying information was removed during transcript preparation. Quotations reported in the manuscript were translated from Chinese into English and carefully checked against the original Chinese transcripts to preserve their intended meaning.

Data collection and analysis proceeded iteratively. As the analysis progressed beyond approximately 20 interviews, the major categories and their relationships became increasingly stable, while subsequent interviews yielded largely repetitive rather than theoretically novel information. The primary category structure and relational model were developed through the analysis of 24 interviews. The remaining six interviews were then analyzed sequentially as additional cases to assess whether further data would generate theoretically consequential categories or require modification of the emerging model. The procedures and criteria used to assess theoretical saturation are described in Section 3.5 (Table 1).

**Table 1:** Participant characteristics (*N* = 30).

Characteristics	Category	*n*	%
Gender			
	Female	14	46.67
	Male	16	53.33
Year of study			
	Freshman	7	23.33
	Sophomore	8	26.67
	Junior	8	26.67
	Senior	7	23.33
Major			
	Social sciences	12	40.00
	Natural sciences	10	33.33
	Humanities and arts	8	26.67
Region of China			
	Eastern	11	36.67
	Central	9	30.00
	Western	10	33.33

Participants ranged in age from 18 to 25 years.

### 3.3 Interview protocol

The semi-structured interview protocol was designed to elicit participants’ experiences rather than responses to predetermined theoretical categories. Questions focused on three broad domains: (a) students’ experiences and challenges in online learning; (b) how they planned, monitored, adjusted, and reflected on their learning; and (c) how teacher-student interaction and feedback influenced, or failed to influence, these regulatory processes. Follow-up probes encouraged participants to provide concrete examples, describe changes in their learning behavior, and explain what occurred before and after particular interactional events.

The complete interview protocol is provided in Appendix A.

### 3.4 Data analysis and coding procedures

Data analysis followed the iterative procedures of Straussian grounded theory, combining open coding, axial coding, selective coding, and constant comparison. Analysis proceeded alongside repeated comparison of incidents, codes, and categories rather than as a strictly linear sequence (Corbin and Strauss, 2015; Strauss and Corbin, 1998). Both authors participated in the coding process. Coding decisions and category development were discussed iteratively, and disagreements were resolved through discussion (Figure 1).

**Figure 1:**
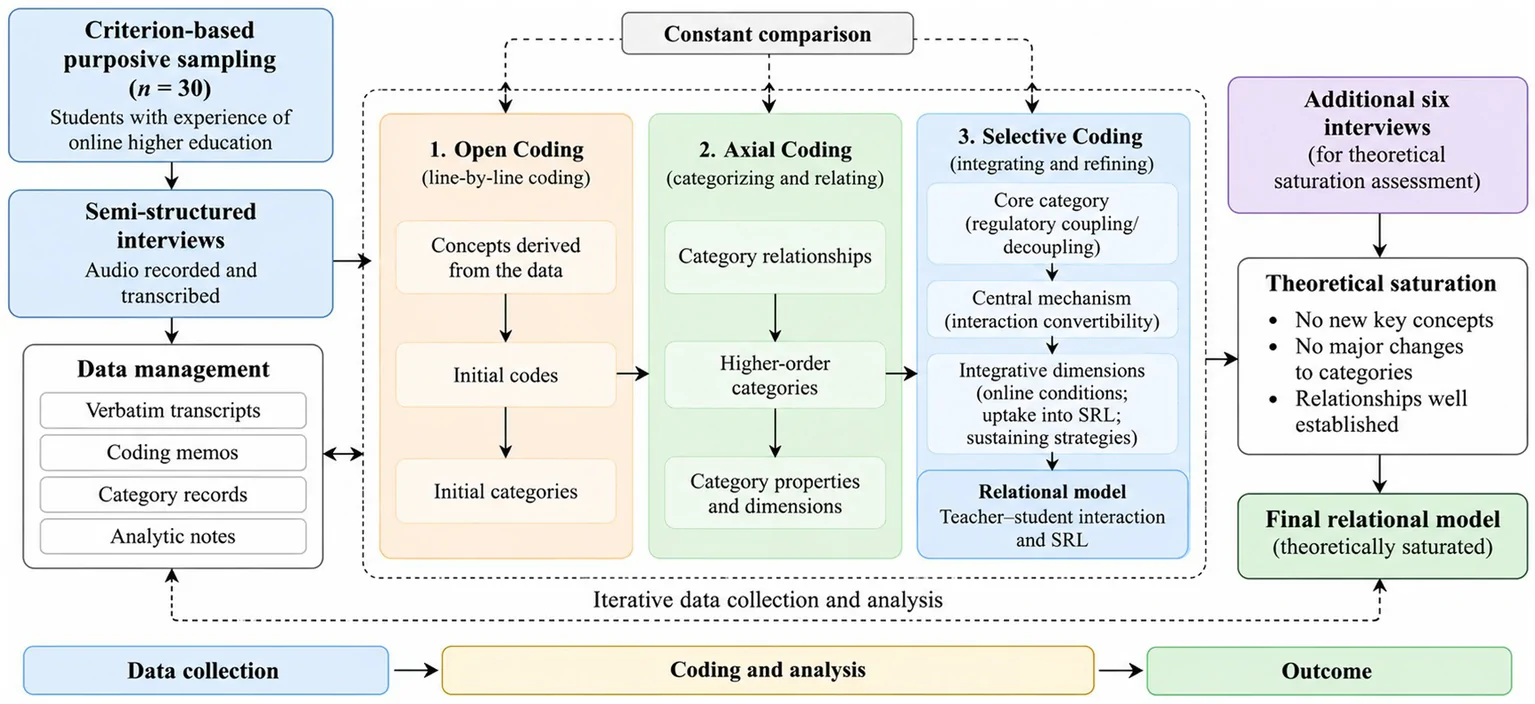
Procedure of the grounded theory analysis.

During open coding, the 24 primary transcripts were examined closely to identify meaning units related to online learning conditions, teacher-student interaction, and students’ regulatory actions. Initial codes remained close to participants’ accounts and captured actions, experiences, or conditions such as attentional refocusing following teacher questioning, correction of misunderstanding after feedback, distraction under reduced monitoring, and follow-up help seeking. Conceptually similar codes were progressively compared and grouped, resulting in 19 initial categories.

During axial coding, relationships among the initial categories were examined using the Straussian conditions-actions or interactions-consequences logic. Constant comparison was conducted both within and across interviews to identify similarities, differences, and negative cases. Particular attention was paid to the conditions under which interaction altered students’ subsequent regulatory actions and to cases in which interaction occurred without producing such changes. Through this process, the initial categories were integrated into five higher-order categories concerning online learning conditions, interaction as regulatory support, interaction breakdown, SRL enactment, and strategies for sustaining regulation.

During selective coding, the higher-order categories were compared and integrated around the core category of regulatory coupling or decoupling between teacher-student interaction and self-regulated learning. The analysis focused on explaining how online learning conditions shaped opportunities for coupling, how interactional input was or was not taken up into learners’ regulatory activity, and how sustaining strategies helped stabilize these processes over time. Comparisons with discrepant and negative cases were used to refine category boundaries and clarify the conditions associated with coupling and decoupling.

Selective coding identified interaction convertibility as the central mechanism linking teacher–student interaction to self-regulation. Three integrative dimensions further organized the model: (1) online learning conditions shaping coupling opportunities, (2) interaction uptake into SRL enactment, and (3) sustaining strategies that stabilize coupling over time. These dimensions organize Sections 4.1–4.3, while their integration into the final relational model is presented in Section 4.4 and Figure 2. A more detailed account of the coding structure is provided in Appendix B.

**Figure 2:**
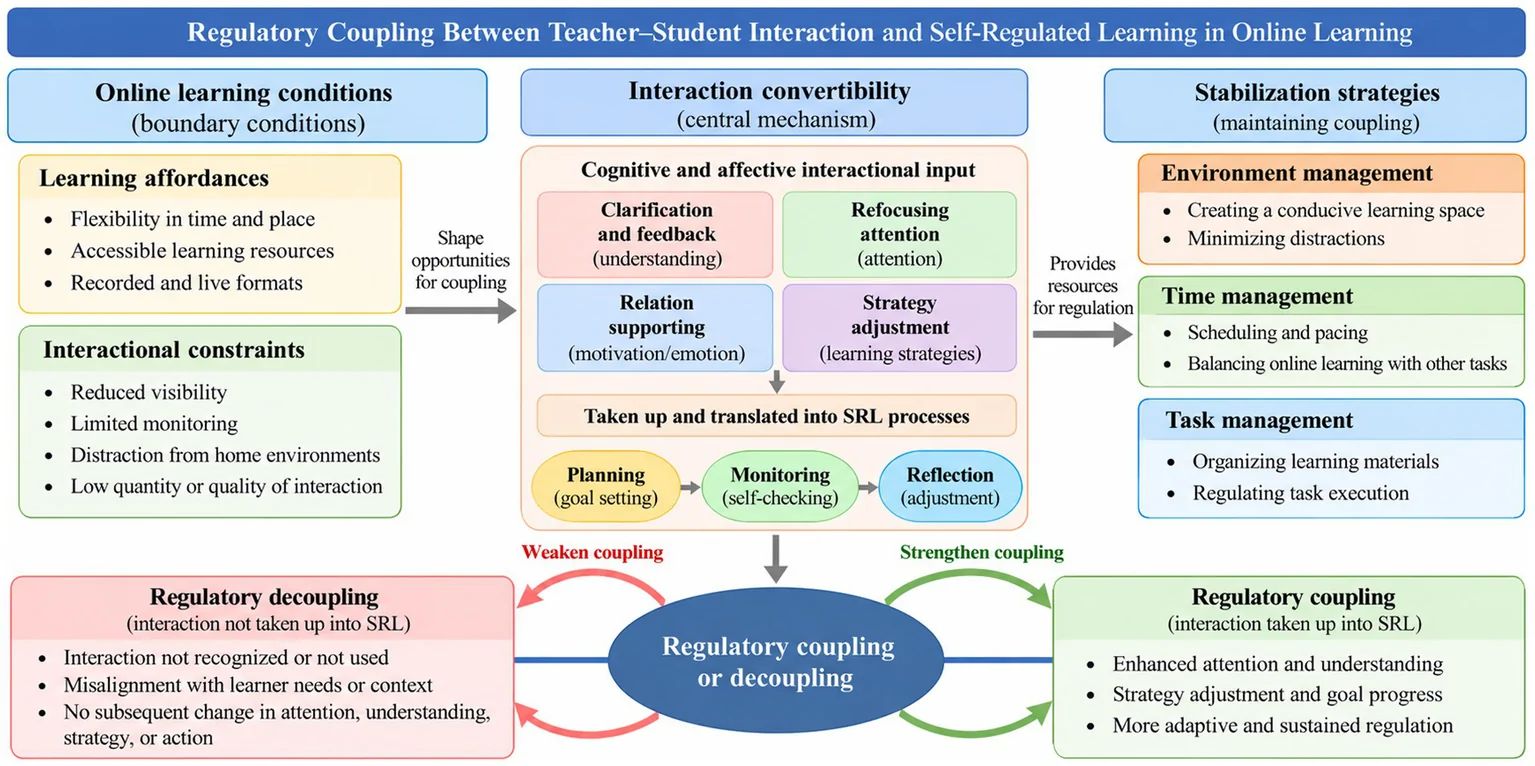
A relational model of regulatory coupling and decoupling.

Constant comparison continued throughout all three stages. Newly identified incidents were compared with previously coded incidents and emerging categories; earlier transcripts were revisited when category definitions changed; and emerging relationships were examined across participants rather than inferred from isolated statements. The final relational model was developed only after the main categories and their relationships had become sufficiently stable across the primary analytic corpus (Table 2).

**Table 2:** Illustrative analytical trail from interview data to the relational model.

Interview excerpt	Initial code	Higher-order category	Relational interpretation
“When the teacher asks questions, my attention will be more concentrated… I will adjust my learning state and keep pace with the teacher”. (Participant d)	Attention redirected through teacher questioning	Interaction as regulatory support	Interaction is taken up into monitoring and adjustment → regulatory coupling
“The teacher may only focus on questions that most people have, but my own question may still remain unresolved”. (Participant p)	Individual need remains unresolved	Interaction breakdown	Interaction occurs but does not generate regulatory adjustment → decoupling
“If I still have questions after class, I write them down and contact the teacher later”. (Participant e)	Follow-up help seeking	SRL enactment/interaction uptake	Unresolved difficulty triggers further regulation and interaction → coupling

### 3.5 Theoretical saturation

Theoretical saturation was assessed because grounded theory requires the developing categories and their relationships to be sufficiently elaborated before the theoretical model is considered complete (Corbin and Strauss, 2015). In this study, saturation assessment was used to determine whether additional interviews would generate theoretically consequential categories, introduce new properties or dimensions, or require substantive revision of the emerging relational model. Theoretical saturation was therefore assessed iteratively during data analysis rather than determined solely by a predetermined sample size (Hennink and Kaiser, 2022). As coding progressed beyond approximately 20 interviews, newly analyzed interviews increasingly reproduced existing patterns, and the main categories and their relationships became progressively stable. The analysis of 24 interviews resulted in a sufficiently developed category structure and preliminary relational model.

The remaining six interviews were subsequently analyzed one by one to assess whether additional data would alter this emerging theoretical structure. Saturation was evaluated using three criteria: whether an additional interview (a) generated a new theoretically consequential category, (b) introduced a new property or dimension that substantively changed an existing category, or (c) revealed a relationship that required revision of the emerging relational model (Corbin and Strauss, 2015; Hennink and Kaiser, 2022). The additional interviews provided further examples and variation within existing categories but did not generate new higher-order categories or require substantive modification of the relationships among them. The category system and relational model were therefore considered theoretically saturated.

## 4 Findings

### 4.1 Online learning conditions: opportunities and risks for regulatory coupling

Participants described online learning as a regulatory environment characterized by both affordances and constraints. These conditions did not determine coupling or decoupling directly. Rather, they shaped the circumstances under which students could regulate their learning and under which teacher-student interaction could become available, timely, and usable. In this sense, online learning conditions functioned as boundary conditions for the relationship between interaction and SRL.

On the enabling side, participants emphasized the structural clarity, pacing flexibility, and resource accessibility of online learning. Participant b described online courses as easier to navigate because of their clearer organization:

*“The content of online courses is clearly presented, so I can easily understand the structure of the lesson.” (Participant b).*

For some learners, this clarity reduced uncertainty about what to attend to and how to organize learning activities. Flexibility in pacing was also valued. Participant f explained:

*“Online courses can be divided into recorded ones and live-streaming ones. Both give me a chance to arrange my study steps, no matter where and when I am.” (Participant f).*

This flexibility enabled learners to adjust the timing and pace of study to their own schedules and learning states. Online environments also expanded access to supplementary resources and enabled immediate responses to gaps in understanding. Participant q noted:

*“During online courses, if I come across some problems, I can check them with the help of the Internet directly. There are so many related materials online.” (Participant q).*

Together, these accounts show that online learning could support regulation by giving learners greater control over pacing, resource selection, and immediate problem solving. Such affordances increased opportunities for self-initiated regulatory action, particularly when learners were able to recognize difficulties and mobilize appropriate resources.

The same environment, however, also weakened some of the conditions through which interaction could support regulation. A recurrent concern was reduced instructor visibility. Participant e explained:

*“In the online course, the teacher can’t see our expressions and learning state, so the teacher can’t give timely and sufficient feedback.” (Participant e).*

Reduced visibility limited instructors’ ability to recognize disengagement or misunderstanding as it occurred, thereby decreasing opportunities for timely corrective interaction. Participant d described the corresponding reduction in external monitoring:

*“In the online class, everyone turns off the video, so the teacher can’t see what I’m doing, and sometimes I just do my other things.” (Participant d).*

Here, the online environment simultaneously reduced teacher monitoring and increased opportunities for off-task behavior. The problem was therefore not simply distraction itself, but the weakening of the interactional cues through which distraction might otherwise be detected and addressed.

Participants also described online learning as vulnerable to shallow engagement. Participant p stated:

*“My understanding of the knowledge points is very shallow, and I have no motivation to carry out in-depth understanding and exploration. I just listen to the course and finish the homework.” (Participant p).*

This account illustrates how reduced monitoring and weaker engagement could narrow learning to task completion without deeper processing. At the same time, some participants experienced a broader scarcity of interaction. Participant f summarized this concern directly:

*“The biggest problem with online learning, I think, is interaction. Face-to-face communication has become a dream.” (Participant f).*

These constraints mattered because interactional support can only enter SRL when learners and instructors have sufficient opportunities to recognize learning problems, communicate about them, and respond in ways that can be acted upon. Reduced visibility, weak monitoring, shallow engagement, and interaction scarcity therefore created conditions under which such regulatory exchange became less likely.

To sum up, the findings indicate that online learning conditions shape the opportunity structure for regulatory coupling. Structural clarity, flexible pacing, and resource accessibility can facilitate self-initiated regulation, while reduced visibility, weak monitoring, distraction, and interaction scarcity constrain the availability and timeliness of external regulatory support. These conditions do not themselves produce coupling or decoupling; instead, they alter the likelihood that interactional input will become available and usable within learners’ regulatory processes. Whether that input is actually converted into SRL depends on the uptake process examined in the next finding.

### 4.2 Interaction convertibility: how interaction enters or fails to enter SRL

Across participants’ accounts, the regulatory value of teacher-student interaction depended less on whether interaction occurred than on what learners subsequently did with the interactional input. We use interaction convertibility to describe the process through which interactional input becomes usable regulatory input and is translated into a change in learners’ attention, understanding, strategy, or subsequent learning action. Regulatory coupling occurs when such input becomes functionally incorporated into learners’ self-regulated learning processes. Decoupling, by contrast, occurs when interaction is present but remains external to regulation and produces little or no consequential change in how learners monitor, interpret, or adjust their learning. These concepts were not imposed as *a priori* coding categories; they emerged through comparison between cases in which interaction altered subsequent regulation and cases in which interaction occurred without such change.

One pathway to regulatory coupling involved attentional refocusing. Participant d described how teacher questioning interrupted disengagement and triggered immediate adjustment:

*“When the teacher asks questions, my attention will be more concentrated, and I will quickly adjust my learning state to keep up with the pace of the teacher’s lectures and learn more efficiently.” (Participant d).*

The teacher’s question did not constitute regulation by itself. Its regulatory value arose because the learner responded by noticing a lapse in attention and adjusting the current learning state. In this case, the interactional sequence can be represented as teacher questioning → attentional refocusing → monitoring and state adjustment. Interaction therefore became integrated into the learner’s ongoing regulation.

A second pathway involved corrective uptake. Participant k recalled:

*“When I answer questions, sometimes I may give a wrong understanding. When the teacher listens to my answer, he can correct my mistakes in time, so that I can correct my original misunderstanding.” (Participant k).*

This account shows a complete transition from interactional feedback to regulatory change: a misunderstanding was made visible, corrective feedback was received, and the learner revised the prior interpretation. The consequential element was therefore not simply the provision of feedback, but the learner’s uptake of that feedback. Here, interaction became coupled with SRL through response → correction → recognition of error → cognitive revision.

A third pathway was extended help seeking, in which learners themselves prolonged the interaction when an unresolved problem remained. Participant e explained:

*“If the time is not enough, I will write down my questions. After class, I will try to get in touch with teachers to discuss these problems.” (Participant e).*

This case highlights learner agency in the coupling process. The learner first identified and recorded an unresolved difficulty and then initiated further interaction after class. Interaction was therefore embedded within a continuing cycle of problem identification and help seeking rather than confined to a single classroom episode. Coupling in this case depended on the learner’s active effort to carry an unresolved regulatory need forward into subsequent interaction.

The contrasting cases show that interaction was not inherently convertible. One form of decoupling arose from need-response mismatch. Participant p described an interactional environment in which teacher responses were available but did not correspond to the learner’s particular difficulty:

*“The teacher may only focus on the questions that most people have, but I may have doubts about other aspects, and the teacher will not focus on this.” (Participant p).*

Here, interaction existed at the class level, but the learner’s regulatory need remained unresolved. Because the available response did not address the specific difficulty, the interaction generated no corresponding correction or strategy adjustment. Interaction therefore remained external to the learner’s regulation.

A second form of decoupling involved low-value repetition. Participant m stated:

*“Some teacher-student interaction is really boring. Without anything new, some teachers repeatedly ask one question one by one, which is of low efficiency.” (Participant m).*

This case distinguishes interaction frequency from regulatory usefulness. Repeated questioning increased the observable amount of interaction but provided little new information for diagnosing understanding, correcting errors, or adjusting learning strategies. The interaction was therefore present without becoming a usable regulatory resource.

A third form of decoupling occurred when interaction became grade-oriented compliance. Participant t explained:

*“I don’t like interaction in some sense, because it has become a method of gaining credits. Only by answering questions actively will teachers give you usual grades.” (Participant t).*

This account separates behavioral participation from regulatory engagement. The student participated in the interaction, but the immediate purpose was to satisfy an assessment requirement rather than to clarify understanding, identify a learning problem, or revise a strategy. Observable participation therefore coexisted with little evidence of regulatory uptake.

Across these cases, interaction convertibility can be understood as a three-part process: (1) interaction provides regulation-relevant input; (2) the learner recognizes and takes up that input; and (3) the input is translated into a consequential change in attention, understanding, strategy, or subsequent action. Regulatory coupling occurs when this translation connects interaction to learners’ ongoing regulatory activity. Decoupling occurs when one or more links in this process are interrupted—for example, when interaction does not correspond to the learner’s need, provides little usable information, or elicits participation without substantive learning uptake. The distinction between coupling and decoupling therefore lies in the regulatory consequence of interaction, rather than in its occurrence or frequency alone.

To make these contrasting pathways explicit, Table 3 summarizes how different forms of interactional input were either incorporated into SRL or remained disconnected from it.

**Table 3:** Empirical pathways from interactional input to regulatory coupling and decoupling.

Empirical pathway	Interactional input	Learner uptake/non-uptake	Regulatory consequence	Relational outcome	Illustrative case
Attentional refocusing	Teacher questioning	Attention redirected to the learning task	Monitoring and learning-state adjustment	Coupling	Participant d
Corrective uptake	Teacher correction	Feedback recognized and acted upon	Cognitive revision	Coupling	Participant k
Extended help seeking	Unresolved question	Learner initiates follow-up interaction	Continued problem solving	Coupling	Participant e
Need-response mismatch	Teacher addresses majority concerns	Individual need remains unresolved	No corrective adjustment	Decoupling	Participant p
Low-value repetition	Repetitive questioning	Interaction provides little usable information	Limited regulatory adjustment	Decoupling	Participant m
Grade-oriented compliance	Participation tied to grades	Behavioral participation without substantive uptake	Little or no regulatory change	Decoupling	Participant t

### 4.3 Sustaining strategies and the stabilization of regulatory coupling

A third finding concerns how learners sustained regulation after interactional support had become available and usable. Participants described a range of practices that helped maintain attention, learning progress, and continuity across learning episodes. These practices did not create regulatory coupling by themselves. Rather, they functioned as stabilizing conditions that enabled learners to remain sufficiently engaged to recognize, use, and act on interactional input over time.

One form of stabilization involved the deliberate mobilization of external resources. Participants used digital tools, learning materials, and social relationships to reduce distraction and support continued regulation. Participant f described using learning applications to protect attention:

*“Self-regulated learning is the key to keeping attentive in online courses. Therefore, I will make use of some study apps to keep myself away from entertainment.” (Participant f).*

Other participants combined digital resources with conventional learning aids. Participant l explained:

*“Notes, mind maps, and exercise books help me gain a good command of the lessons. Moreover, I will look for Internet resources such as MOOCs.” (Participant l).*

Social support was incorporated in a similar way. When learning problems persisted, students sought assistance from peers rather than relying solely on individual regulation. Participant h stated:

*“Most of the time, when I come across some troubles in learning, especially during online courses, I will send WeChat messages to my friends or classmates first. They are willing to help me solve these problems and give me lots of inspiration and ideas.” (Participant h).*

These accounts show that online SRL was sustained through resource mobilization as well as internal self-control. Tools, materials, and social support became part of the regulatory environment when learners actively incorporated them into ongoing learning activity.

A second form of stabilization involved maintaining continuity within the regulatory cycle itself. Participants described monitoring and reflection as practices that helped them preserve awareness of their learning state and identify when adjustment was needed. Participant i described in-class monitoring:

*“During online class, I will keep highly focused on what the teacher has said, take notes, highlight key points, and, in a word, monitor myself.” (Participant i).*

Participant k emphasized post-class reflection:

*“After online courses, self-reflection and self-conclusion are useful for helping us review what level we have reached.” (Participant k).*

These practices provided a regulatory structure into which interactional input could be incorporated. Feedback or clarification received during learning was more likely to become consequential when learners were already monitoring their understanding and later revisiting unresolved problems. In this sense, coupling was easier to sustain when interaction could be connected to an ongoing sequence of monitoring, evaluation, and adjustment rather than remaining an isolated event.

A third form of stabilization concerned the management of environment, time, and tasks. Participants described environmental control as a way to protect attention from the distraction risks of online learning. Participant d explained:

*“To improve online self-regulated learning abilities, it is important to find a peaceful environment without entertainment and distractions.” (Participant d).*

Time management served a similar function by maintaining continuity in learning progress. Participant p noted:

*“We have to keep up with the progress of the online course. If we don’t listen to teachers carefully, it may take more time to make up after class, and the effect may not be as good as listening to the teacher’s explanation.” (Participant p).*

Task prioritization helped learners manage competing demands:

*“Under the online environment, lots of tasks are individual. Therefore, we need to identify which task is the most important or the most difficult.” (Participant q).*

These practices stabilized the conditions under which regulation could continue. Environmental control reduced attentional disruption, time management helped preserve continuity between learning episodes, and task prioritization reduced the likelihood that competing demands would displace ongoing learning. Together, they increased the likelihood that learners would remain engaged enough to recognize relevant interactional support and translate it into subsequent action.

Participants also emphasized that productive interaction should extend across learning phases rather than remain confined to the synchronous class session. Participant c stated:

*“Teacher-student interaction is not only limited to class. It should include communication before class and discussion after class.” (Participant c).*

Participant i similarly described interaction as cyclical:

*“Interaction should be a circle. For online learning, this kind of interaction is especially important.” (Participant i).*

These accounts suggest that sustained regulatory coupling is more likely when interaction can recur across pre-class preparation, in-class monitoring, and post-class reflection. Stable self-regulation enables learners to carry questions, feedback, and unresolved problems from one phase of learning into the next, allowing interaction to function as part of a continuing regulatory process rather than as a one-off exchange.

In conclusion, the findings indicate that regulatory coupling requires more than effective interaction at a single moment. Resource mobilization, continuity of monitoring and reflection, and the management of environment, time, and tasks help preserve the regulatory conditions under which interaction can continue to be recognized and used. These sustaining strategies therefore function as stabilization mechanisms: they do not generate coupling independently, but they increase the likelihood that coupling, once established, can persist across learning phases.

### 4.4 A relational model of regulatory coupling and decoupling

Integrating the three findings yielded a relational model centered on regulatory coupling between teacher-student interaction and self-regulated learning. Regulatory coupling refers to a relational state in which interactional input becomes incorporated into learners’ ongoing regulatory activity and contributes to changes in attention, understanding, strategy, or subsequent action. Decoupling refers to situations in which interaction and SRL remain functionally disconnected: interaction may occur, but it does not produce consequential regulatory change, or learners’ regulation proceeds with little meaningful support from teacher-student interaction.

The model consists of three interrelated processes. First, online learning conditions shape opportunities for coupling. Structural clarity, flexible pacing, and resource accessibility can support learners’ own regulatory activity, whereas reduced visibility, weak monitoring, distraction, and interaction scarcity constrain opportunities for timely and responsive exchange. These conditions do not determine the relational outcome, but they establish the context within which interaction and regulation unfold.

Second, interaction convertibility constitutes the central mechanism linking interaction with SRL. As shown in Finding 2, interaction becomes regulatory only when learners recognize and take up regulation-relevant input and translate it into changes in their learning. Teacher questioning may redirect attention, corrective feedback may prompt cognitive revision, and unresolved problems may lead to extended help seeking. By contrast, need-response mismatch, repetitive interaction, and grade-oriented participation interrupt this translation process. Coupling therefore depends less on the amount of interaction than on whether interaction becomes usable within learners’ regulatory processes.

Third, sustaining strategies stabilize coupling over time. Resource mobilization, monitoring and reflection, and the management of environment, time, and tasks help learners maintain sufficient regulatory continuity to recognize and use interactional support across different phases of learning. These strategies do not independently create coupling; rather, they increase the likelihood that interaction, once incorporated into SRL, remains consequential beyond a single episode.

Taken together, the model suggests a relational process of boundary conditions → interaction convertibility → stabilization. This sequence should not be interpreted as a rigid or unidirectional causal chain. Participants’ accounts indicate a recursive relationship: effective interaction can support regulation, while active regulation can make learners more likely to identify problems, seek help, and use subsequent feedback. Coupling can therefore become self-reinforcing when responsive interaction and sustained regulation support one another. Conversely, when visibility, responsiveness, learner uptake, or regulatory continuity breaks down, interaction and SRL may become increasingly decoupled.

Figure 2 summarizes this relational structure. Online learning conditions define the opportunity structure within which interaction occurs; interaction convertibility explains whether interactional input enters learners’ SRL; and sustaining strategies help preserve coupling across learning phases. The model therefore shifts attention from whether interaction occurs to how interaction becomes regulatory, under what conditions this conversion succeeds or fails, and how the resulting relationship is sustained over time.

## 5 Discussion

### 5.1 Theoretical contributions

This study contributes to research on online learning and self-regulated learning in three ways. Rather than treating teacher-student interaction and SRL as parallel predictors of learning outcomes, the findings specify how the two become connected through a relational regulatory process.

First, the study conceptualizes online learning as a set of boundary conditions for regulation, rather than simply as an instructional modality. Existing research has documented the flexibility and resource accessibility of online learning alongside challenges such as distraction, reduced visibility, and weaker interaction (van Dorresteijn et al., 2025). The present findings extend this literature by showing that these conditions matter because they shape the opportunity structure within which interaction and self-regulation can become connected. Structural clarity and resource accessibility can support self-initiated regulation, whereas reduced visibility, weak monitoring, and interaction scarcity constrain opportunities for timely regulatory support. Online learning conditions therefore influence not only whether students can regulate their learning, but also whether interactional support is sufficiently available and responsive to enter that regulatory process. This shifts attention from comparing online and offline environments as fixed formats toward examining the specific conditions under which regulatory relationships become more or less viable.

Second, the study develops interaction convertibility as a mechanism linking teacher-student interaction with SRL. This finding extends longstanding accounts of feedback as integral to self-regulation (Butler and Winne, 1995; Nicol and Macfarlane-Dick, 2006) and more recent work emphasizing learners’ feedback uptake and responses to feedback (Carless and Young, 2025; Yu et al., 2026). The present study extends this uptake perspective beyond feedback alone by showing that a broader range of interactional inputs, including questioning, clarification, correction, and help seeking, become regulatory only when they are translated into subsequent learning action. In the present data, such conversion was evident when interaction prompted attentional refocusing, cognitive revision, or continued problem solving, whereas need-response mismatch, low-value repetition, and grade-oriented participation disrupted this process. Interaction convertibility therefore shifts attention from the provision or frequency of interaction to the process through which interaction becomes usable within learners’ regulatory activity.

This mechanism also helps clarify the distinction between regulatory coupling and decoupling. Coupling occurs when interaction becomes functionally incorporated into learners’ SRL, whereas decoupling occurs when interaction remains present but produces little consequential regulatory change. This distinction is theoretically useful because it explains why apparently similar levels of interaction may have different learning consequences. Interaction should therefore not be assumed to exert a uniform positive effect; its contribution depends on the learner’s uptake of regulation-relevant input and on the fit between that input and the learner’s current regulatory needs.

Third, the study extends the conceptualization of SRL by emphasizing regulatory stabilization. SRL is often described in terms of planning, monitoring, and reflection, but the findings show that sustaining these processes in online learning also depends on learners’ ability to mobilize resources and preserve regulatory continuity (Dong et al., 2024; Zhao et al., 2025). Digital tools, supplementary materials, peer support, environmental control, time management, and task prioritization helped learners maintain attention and progress across learning episodes. These practices mattered not only because they supported individual regulation, but because they increased the likelihood that learners could recognize and use interactional support across pre-class, in-class, and post-class phases. SRL in online learning therefore emerges as a system involving regulatory enactment, external resource mobilization, and stabilization over time.

### 5.2 Practical implications

The findings suggest that improving online learning requires attention not simply to the amount of interaction provided, but to whether interaction can be recognized, used, and translated into regulatory action. For instructors and course designers, this means prioritizing interaction that is timely, diagnostically useful, and responsive to learners’ actual difficulties (Zhang et al., 2026). Brief comprehension checks, structured question channels, and opportunities for individualized follow-up can help instructors identify misunderstandings that may remain invisible in online settings. At the same time, interaction should avoid becoming repetitive or primarily compliance-driven. When participation is organized mainly around frequency or grades, students may participate behaviorally without using the interaction to monitor understanding or adjust learning strategies. Instructional design should therefore emphasize the usefulness of interaction rather than its sheer quantity.

The findings also indicate that interaction is more likely to support SRL when it is distributed across learning phases. Pre-class interaction can clarify expectations and learning goals, in-class interaction can help identify and correct emerging difficulties, and post-class communication can support reflection and unresolved problem solving. Designing interaction across these phases may make it easier for students to connect teacher input with planning, monitoring, and reflection rather than treating interaction as an isolated classroom event. This is consistent with formative-feedback perspectives that emphasize feedback as information for monitoring and subsequent adjustment rather than as an endpoint in itself (Nicol and Macfarlane-Dick, 2006). This implication follows directly from participants’ descriptions of interaction as a continuing process extending beyond the synchronous lesson.

For students, the findings highlight the importance of maintaining the regulatory conditions required to benefit from interaction. Environment management, time management, and task prioritization can help protect attention and preserve learning continuity in distraction-prone online settings. Equally important, students may benefit from treating feedback and interaction as inputs for subsequent action, for example, by identifying unresolved questions, revising misunderstandings, updating learning plans, or seeking further clarification when necessary. In this sense, effective self-regulation involves not only managing one’s own behavior but also making purposeful use of available instructional and social resources.

### 5.3 Limitations and future research

Several limitations should be considered when interpreting the findings. First, the study relies on self-reported and retrospective interview data. Participants described their learning and interaction experiences after they had occurred, and these accounts may therefore be influenced by recall bias, selective reconstruction, or social desirability. Interview narratives also capture how students understood and interpreted their regulatory behavior rather than providing direct evidence of what they actually did at each moment of learning. Future research could combine interviews with behavioral indicators such as learning-platform logs, recorded interaction episodes, stimulated-recall data, or other process measures to examine whether reported patterns of feedback uptake and regulatory adjustment are reflected in observable learning behavior.

Second, the findings are situated within the context of Chinese university students. Teacher-student relationships, expectations regarding classroom participation, feedback practices, assessment arrangements, and the organization of online learning may differ across educational and cultural settings. The proposed mechanisms of interaction convertibility and regulatory coupling should therefore be interpreted as theoretically grounded within this context rather than assumed to generalize directly to other higher education systems. Comparative studies across countries, institutions, course formats, and disciplinary contexts could help clarify which elements of the model are context-specific and which are more broadly transferable.

Third, the analysis primarily reflects the learner perspective. Although students could describe whether interaction was useful to them, the study did not directly examine instructors’ intentions, instructional decisions, workload constraints, or perceptions of the same interactional episodes. As a result, instances of decoupling cannot always be attributed precisely to instructional design, interaction enactment, learner uptake, or the fit between them. Future research incorporating instructor interviews, classroom observations, and interaction trace data could provide a more complete account of where and why regulatory coupling succeeds or breaks down.

Finally, the relational model developed here is an interpretive and theory-building model rather than a statistically validated causal model. Concepts such as interaction convertibility and regulatory coupling emerged through grounded analysis and require further operationalization and testing. Future studies could develop measures of these constructs and examine their temporal and directional relationships through longitudinal, mixed-method, or experimental designs. Such work would help determine the robustness of the proposed mechanisms and further specify the conditions under which interaction becomes consequential for SRL.

## 6 Conclusion

This study developed a relational model explaining how teacher-student interaction becomes connected with self-regulated learning in online higher education. Drawing on the experiences of 30 Chinese university students, the analysis shows that this relationship cannot be understood simply in terms of whether interaction occurs or how frequently students interact with instructors. Its regulatory significance depends on the conditions under which interaction occurs, whether learners take up interactional input, and whether regulation can be sustained over time.

Three conclusions follow. First, online learning conditions establish the boundary conditions for interaction and regulation. Structural clarity, flexible pacing, and accessible resources can support self-initiated regulation, while reduced visibility, weak monitoring, distraction, and interaction scarcity constrain opportunities for timely regulatory support. Second, interaction convertibility constitutes the central mechanism linking interaction with SRL. Interaction becomes regulatory when learners recognize and use interactional input to refocus attention, revise understanding, seek further help, or adjust subsequent action. When interaction is mismatched to learner needs, repetitive, or primarily compliance-oriented, it may remain functionally disconnected from regulation, producing decoupling. Third, resource mobilization, monitoring and reflection, and the management of environment, time, and tasks help stabilize regulatory coupling across learning phases.

The resulting model therefore shifts attention from the quantity of interaction to its regulatory consequences. Teacher-student interaction becomes educationally consequential when it can be converted into a usable resource within learners’ ongoing regulatory activity. By specifying the conditions, uptake processes, and sustaining mechanisms involved in this relationship, the study provides a process-oriented account of how interaction and SRL become coupled or decoupled in online higher education.

## Data Availability

The raw interview data are not publicly available because they contain potentially identifiable contextual information and the participants did not consent to public sharing of their full interview transcripts. De-identified excerpts supporting the findings are included in the article and Supplementary material.
