# Supplementary material for: Teacher-student interaction and self-regulated learning in online higher education: a grounded theory model of interaction convertibility
Source: Front Psychol. 2026 Oct 13;17:1989345. doi: 10.3389/fpsyg.2026.1989345 (PMC13710346; doi:10.3389/fpsyg.2026.1989345)
Supplement: Supplementary file 1 [file Supplementary_file_1.docx]

**Appendix A. Semi-Structured Interview Protocol**

The interviews followed a semi-structured protocol organized around participants’ online learning experiences, self-regulated learning practices, and teacher-student interaction. The questions below served as the core interview guide. Their order could be adjusted according to the flow of the interview, and follow-up probes were used when necessary to elicit concrete experiences, examples, and changes in learning behavior.

**Q1.** How do you understand self-regulated learning in your own words? What behaviors or abilities best represent it in online learning?

**Q2.** What kinds of online courses have you taken, and how would you evaluate your learning experience?

**Q3.** During online courses, how did you plan, monitor, and adjust your learning? Can you recall a specific example?

**Q4.** What forms of teacher-student interaction did you experience, such as question-and-answer exchanges, feedback, discussions, or reminders? How often did these interactions occur, and how useful did you find them?

**Q5.** Please describe a memorable interaction with an instructor. How did it influence your learning strategies, motivation, or management of learning progress?

**Q6.** Based on your experience, what kinds of support would help students strengthen their self-regulated learning in online courses? What could instructors or online learning platforms do differently?
