# Supplementary material for: Teacher-student interaction and self-regulated learning in online higher education: a grounded theory model of interaction convertibility
Source: Front Psychol. 2026 Oct 13;17:1989345. doi: 10.3389/fpsyg.2026.1989345 (PMC13710346; doi:10.3389/fpsyg.2026.1989345)
Supplement: Supplementary file 2 [file Supplementary_file_2.docx]

Appendix B. Coding Framework and Category Development

*Note. This appendix presents a summarized coding framework used in the primary analysis. It illustrates the progression from data-near codes to initial categories, higher-order categories, and selective integration; it does not reproduce the complete line-by-line coding record. Ellipses indicate omitted wording, and participant/segment identifiers are retained from the original coding record.*

**Table B1. Open-Coding Framework: Initial Categories, Representative Codes, and Illustrative Excerpts**

| **Empirical domain** | **Initial category** | **Representative initial codes** | **Illustrative participant excerpts** |
| --- | --- | --- | --- |
| **Online learning conditions** | 1. Clear structure and concise delivery | Concise explanation; focused key points; clear lesson structure/logic | a2: “Online courses are focused on knowledge points with direct illustration.” / h1: “There are no redundant and tedious explanations in online courses.” / b2: “The contents of online courses are clearly presented, so I can easily understand the structure of the lesson.” |
|  | 2. Flexible pacing and manageable duration | Flexible scheduling; recorded/live formats support pacing; appropriate session length and breaks | c1: “We can choose the suitable time to learn the online courses; it is convenient.” / f2: “Recorded and live-streaming courses give me a chance to arrange my study steps, no matter where and when I am.” / m3: “The course length gives me enough break time to find my best study state.” |
|  | 3. Supplementary resources and just-in-time information search | Pre-uploaded materials enable preview; teacher-provided resources; searching online when stuck | p2: “Teaching materials are uploaded ahead, so we can preview.” / g2: “Teachers offer relevant resources, including documentaries and learning websites.” / q3: “If I come across problems, I can check them on the Internet directly.” |
|  | 4. Limited instructional variety and reduced feedback/visibility | PPT-reading delivery; teacher cannot see students’ learning state; feedback not timely/sufficient | r3: “Most teachers just read the PPT; I feel tired trying to pay attention.” / e2: “The teacher can’t see our expression and learning state, so the teacher can’t give timely and sufficient feedback.” |
|  | 5. Low monitoring and increased off-task risk | Cameras off reduce monitoring; learners multitask during class | d1: “Everyone turns off the video, so the teacher can’t see what I’m doing, and sometimes I just do my other things.” |
|  | 6. Shallow processing, distraction, and interaction scarcity | Superficial understanding; home/dorm distractions; interaction constrained relative to face-to-face learning | p1: “My understanding is very shallow… I just listen and finish the homework.” / o2: “During online classes, I often lose my mind… family members suddenly enter.” / f3: “The biggest problem is interaction. Face-to-face communication has become a dream.” / q4: “Teacher–student interaction… and peer communication are limited.” |
| **Teacher–student interaction** | 7. Perceived regulatory functions of interaction | Clarify learning status and difficulties; trigger attention and pace adjustment; provide relational closeness; correct misunderstandings | a5: “Interaction helps us understand the learning situation, solve difficult problems, and promote deeper understanding.” / d6: “When the teacher asks questions, my attention becomes more concentrated… I adjust my learning state.” / h5: “Face-to-face interaction gives a sense of closeness.” / k6: “If my understanding is wrong, the teacher can correct it.” |
|  | 8. Interaction quality problems: delay, mismatch, low efficiency, and instrumental participation | Interaction disrupts progress; teacher focuses on majority concerns; repetitive/boring interaction; participation for grades | g6: “Interaction should aim at learning effects, but sometimes it affects progress.” / p8: “The teacher may only focus on questions that most people have, but I may have other doubts.” / m7: “Some interaction is really boring… repeatedly asking one question.” / t5: “Interaction has become a method of gaining credits… answering questions actively gives you usual grades.” |
|  | 9. Interaction across learning phases | Pre-class communication; in-class interaction; post-class discussion; interaction as a cycle | c6: “Interaction is not only limited to class; it should include communication before class and discussion after class.” / i7: “Interaction should be a circle.” |
|  | 10. Interaction formats and pedagogical moves | Questioning; debating; exemplifying; critiquing/correcting; role-playing | k6: “Teacher asks questions… students answer… teacher criticizes and corrects.” / q7: “Teachers give examples, ask students to debate, and finally conclude.” / j5: “Role play… can present the problem and make us think more deeply.” |
|  | 11. Enabling and constraining conditions of interaction | Network stability; content attractiveness; teaching-method diversity; student engagement state | o8: “Network conditions matter… if I can’t hear questions clearly, the teacher will not receive my response.” / s5: “Attractive content and diverse methods increase enthusiasm.” / t6: “If students remain highly engaged, the effect is good; if absent-minded, the effect is reduced.” |
| **Self-regulated learning** | 12. Digital tools and learning aids for self-regulation | Devices; learning apps; platform services; notes/mind maps; practice questions; external online resources | a9: “I use different electronic products, such as an iPad.” / f10: “I use study apps to keep me away from entertainment.” / l9: “Notes, mind maps, and exercise books… and Internet resources such as MOOCs.” |
|  | 13. Social support as regulatory resources | Teacher help-seeking after class; peer discussion via messaging; parental support for the study environment | e9: “I write down my questions… after class I get in touch with teachers.” / h10: “I send WeChat messages to my friends.” / k12: “My parents create a quiet study environment and prepare food.” |
|  | 14. Affect, rhythm, mindset, and self-efficacy in SRL | Emotion regulation; finding a suitable learning rhythm; learning purpose/mindset; self-efficacy | e9: “SRL is closely linked with lifestyle, mindset, and emotion.” / m10: “We readjust our learning rhythm.” / x9: “The purpose is to learn more efficiently… adjust the learning state and strategy.” |
|  | 15. Planning–monitoring–reflection cycle | Preview/forethought; in-class monitoring through notes/highlighting; post-class review and summarizing | b10: “Preview is extremely important.” / i11: “I keep highly focused, take notes, highlight key points, and monitor myself.” / k9: “Self-reflection and self-conclusion help me review.” |
|  | 16. Reflection, doubt-solving, and self-evaluation | Solving doubts; reflection and evaluation; adjusting subsequent learning | c9: “The ability to solve doubts.” / j9: “Self-reflection and evaluation… then adjust my self-learning.” |
|  | 17. Environment management and distraction control | Ritualization; quiet atmosphere; entertainment control | n9: “A sense of ritual… dressing neatly helps me enter a better state.” / d11: “Find a peaceful environment without entertainment and distractions.” |
|  | 18. Time management and schedule adjustment | Trade-off between study and leisure; balancing regularity and deadlines; keeping pace with course progress | k12: “Change online-learning plans… exchange study time and play time.” / n10: “Balance regularity and finality.” / p10: “Keep up with the progress… otherwise make up after class.” |
|  | 19. Task prioritization under multiple demands | Identify task importance/difficulty; prioritize tasks; orderly completion | q12: “Identify which task is the most important or the most difficult.” / t12: “Give priority… and carry tasks out in an orderly way.” |

**Table B2. Higher-Order Categories Developed Through Axial Coding**

| **Higher-order category** | **Linked initial categories** | **Analytical meaning** |
| --- | --- | --- |
| A. Online learning conditions: affordances and constraints | 1–6 | Online learning offers structural clarity, flexible pacing, and resource accessibility while also reducing visibility, monitoring, and interaction density. These conditions form the boundary context in which interaction and SRL can occur. |
| B. Interaction as regulatory support | 7, 9, 10 | Teacher–student interaction can function as an external regulatory resource by clarifying difficulties, refocusing attention, providing correction, and supporting learning across pre-, in-, and post-class phases. |
| C. Interaction breakdown: mismatch, low value, and formalism | 8, 11 | Interaction may fail to support regulation when it is delayed, poorly matched to individual needs, repetitive or inefficient, technologically constrained, or reduced to grade-oriented participation. |
| D. SRL enactment: resource mobilization and regulatory cycle | 12–16 | Learners enact SRL by mobilizing digital and social resources and by engaging in affect regulation, planning, monitoring, reflection, doubt-solving, and self-evaluation. Interaction becomes consequential when it is taken up within these regulatory activities. |
| E. Sustaining SRL: stabilization strategies | 17–19 | Environment, time, and task management help preserve attention and progress, creating the conditions under which interactional support can continue to be recognized and used across learning phases. |

**Table B3. Selective Integration of the Relational Model**

| **Selective-coding output** | **Concept / dimension** | **Linked axial categories** | **Interpretive role in the relational model** |
| --- | --- | --- | --- |
| Core category | Regulatory coupling / decoupling between teacher–student interaction and SRL | A–E | Captures whether interaction becomes functionally incorporated into learners’ regulatory activity or remains disconnected from it. |
| Central mechanism | Interaction convertibility | B, C, D | Explains whether regulation-relevant interactional input is recognized, taken up, and translated into changes in attention, understanding, strategy, or subsequent action. |
| Integrative dimension 1 | Online learning conditions shaping coupling opportunities | A, C | Affordances and constraints establish the opportunity structure for timely, responsive, and usable interaction. |
| Integrative dimension 2 | Interaction uptake into SRL enactment | B, C, D | Coupling emerges when interactional input is taken up into regulatory processes; decoupling occurs when uptake is interrupted by mismatch, low usefulness, delay, or compliance-oriented participation. |
| Integrative dimension 3 | Sustaining strategies stabilizing coupling over time | E | Environment, time, and task management, together with ongoing monitoring and reflection, help preserve regulatory continuity across learning phases. |
